# Moving beyond the benchmarks: Five foundational principles for meaningful AI evaluation in healthcare

**DOI:** 10.1371/journal.pdig.0001115

**Published:** 2026-05-26

**Authors:** Catherine G. Bielick, Aya Awwad, Jacob Ellen, Laleh Jalilian, Liam G. McCoy, Vishala Mishra, Esli Osmanlliu, Stephen R. Pfohl, Leo A. Celi

**Affiliations:** 1 Division of Infectious Diseases, Beth Israel Deaconess Medical Center, Boston, Massachusetts, United States of America; 2 Harvard Medical School, Boston, Massachusetts, United States of America‌‌; 3 Metabolism Unit, Massachusetts General Hospital, Boston, Massachusetts, United States of America; 4 David Geffen School of Medicine, University of California, Los Angeles, Los Angeles, California, United States of America; 5 Division of Neurology, Faculty of Medicine and Dentistry, University of Alberta, Edmonton, Alberta, Canada; 6 Department of Medicine, Beth Israel Deaconess Medical Center, Boston, Massachusetts, United States of America; 7 Institute for Medical Engineering and Science, Massachusetts Institute of Technology, Cambridge, Massachusetts, United States of America; 8 Duke University School of Medicine, Durham, North Carolina, United States of America; 9 Department of Pediatrics, Division of Pediatric Emergency Medicine, Montreal Children’s Hospital, Montreal, Quebec, Canada; 10 Google Research, Mountain View, California, United States of America; 11 Laboratory for Computational Physiology, Massachusetts Institute of Technology, Cambridge, Massachusetts, United States of America; 12 Division of Pulmonary, Critical Care and Sleep Medicine, Beth Israel Deaconess Medical Center, Boston, Massachusetts, United States of America; 13 Department of Biostatistics, Harvard T.H. Chan School of Public Health, Boston, Massachusetts, United States of America; McGill University Faculty of Science, CANADA

## Abstract

Rapid integration of Large Language Models (LLMs) into healthcare has exposed a critical disconnect between technical performance and clinical value. While state-of-the-art models achieve impressive scores on standardized medical examinations, their real-world impact remains limited, with few models progressing to successful clinical integration. This disconnect persists, in part, due to a proliferation of evaluation practices that prioritize static, decontextualized benchmarks. To help address this gap, we propose five foundational principles to guide contextually appropriate evaluations of healthcare AI: Local (grounded in specific deployment contexts), Task-specific (aligned with intended clinical use), Agile (continuously adaptive), Reflective (acknowledging limitations and inherent value-sensitivity), and Community-partnered (centering affected voices). We argue that emphasis on these principles can help shift evaluation practice towards assessment of artificial intelligence. This reorientation is essential for developing healthcare AI that not only performs well technically, but also can meaningfully improve patient care, serve communities for defined purposes, and mitigate (rather than exacerbate) health disparities.

## Introduction

Modern Large Language Models (LLMs) achieve remarkable performance on medical assessment tasks. GPT-4 demonstrated 80–90% accuracy across all USMLE steps without specialized training [1], while Google’s Med-PaLM 2 reached 86.5% accuracy on similar questions [2]. Despite these technical successes, both LLMs and non-LLM AI have failed to translate effectively into clinical practice due to a number of safety and evaluation challenges [3,4]. In response to this challenge, recent years have seen an explosion of both guidelines for evaluation and datasets for benchmarking the performance of AI systems and models [5,6]. While well-intentioned, this proliferation has not led to clarity. We argue that the sheer volume of competing frameworks is not a sign of a maturing field but a symptom of a foundational crisis that signals a lack of consensus on the first principles of evaluation. The root of this problem is not a deficit of frameworks, but rather a lack of shared principles for what constitutes rigorous evaluation in a complex sociotechnical domain like healthcare.

Current evaluation paradigms prioritize LLM accuracy on medical question banks while largely neglecting algorithmic bias assessment. Notably, only 5% of evaluated LLM studies used real patient data [7]. Additionally, less than 2% of non-LLM healthcare AI models progress beyond the prototyping phase, but even among the FDA-approved AI-enabled medical devices over half (57%) have not been clinically validated [3,8]. Such an approach divorces evaluation from the social realities that determine whether AI serves patients or merely impresses reviewers or investors. Meaningful progress requires stepping back to establish principles that guide contextually appropriate evaluations. Healthcare AI operates in specific clinical environments serving particular populations with unique needs and contextual definitions of benefit. To this end, we propose five core principles of evaluation for both LLMs and non-LLM AI: Local, Task-specific, Agile, Reflective, and Community-partnered (LTARC). The LTARC approach may serve as a lens for better understanding both the current barriers and opportunities for genuine improvement in healthcare AI evaluation.

While we illustrate LTARC principles using LLMs as examples, given their current prominence in healthcare AI discussions and their particular challenges with outcome definition, the framework applies equally to prediction models, classification systems, reinforcement learning approaches, and other AI paradigms. The core principles of continuous learning, transparency, accountability, regulation, and care-centered design are fundamental requirements for any AI system deployed in healthcare settings.

## Limitations of static benchmarking

Current healthcare AI evaluation relies heavily on static benchmarks, which are standardized datasets and tasks designed for cross-system comparisons. While benchmarks track technological progress, their prevalence reflects a fundamental misunderstanding of meaningful clinical assessment [9]. The dominant paradigm’s reliance on decontextualized benchmarks raises critical questions about validity: improved benchmark performance often does not translate to improved clinical utility of an AI system with respect to its intended use [10]. Even benchmarks satisfying criterion validity cannot substitute for context-specific evaluation grounded in actual deployment settings and populations [9].

Medicine is inherently dynamic. Clinical guidelines evolve constantly, treatment protocols shift between institutions, epidemiology drifts over time, and patient populations change. The COVID-19 pandemic exposed this reality; one systematic review of 62 published AI models found none fit for clinical use, largely due to training-test distribution mismatches and absent prospective validation [11]. Beyond overall temporal instability, static benchmarks fail to capture healthcare’s vast sociotechnical complexity, including clinical workflows, team dynamics, and organizational contexts, which profoundly influence real-world model performance.

Most critically, current benchmarking practices systematically exclude those most affected by AI deployment. Patients, communities, and frontline clinicians rarely participate in benchmark design or validation. One systematic review found only 30% of AI implementation studies meaningfully involved patients or community members in evaluation design [12]. This exclusion shapes what gets measured. Benchmarks reflect institutional and developer priorities: diagnostic accuracy, processing speed, and scalability. They inherently ignore patient priorities such as communication quality, cultural sensitivity, preservation of human connection, and alignment with personal values. Benchmarks provide, at best, weak signals about a model’s full potential. They cannot substitute for an evaluation that is grounded in clinical reality, responsive to temporal change, integrated with social context, and inclusive of affected communities. The proposed LTARC principles are a starting point to address these shortcomings by centering evaluation within specific patient populations and intended uses.

Recent stress testing of frontier medical AI models reveals a troubling gap between benchmark performance and clinical readiness. Gu et al. [13] demonstrated that leading systems could generate correct answers even with critical inputs removed while simultaneously failing when subjected to minor prompt variations and produced ‘convincing yet flawed reasoning traces’ when pressed for justification. These findings underscore that aggregate benchmark scores may conceal brittleness under the incomplete, perturbed, or ambiguous conditions routinely encountered in real-world healthcare settings.

## Five foundational principles for context-aware evaluation of healthcare AI

### Local: grounding evaluation in specific contexts

**Fig 1:**
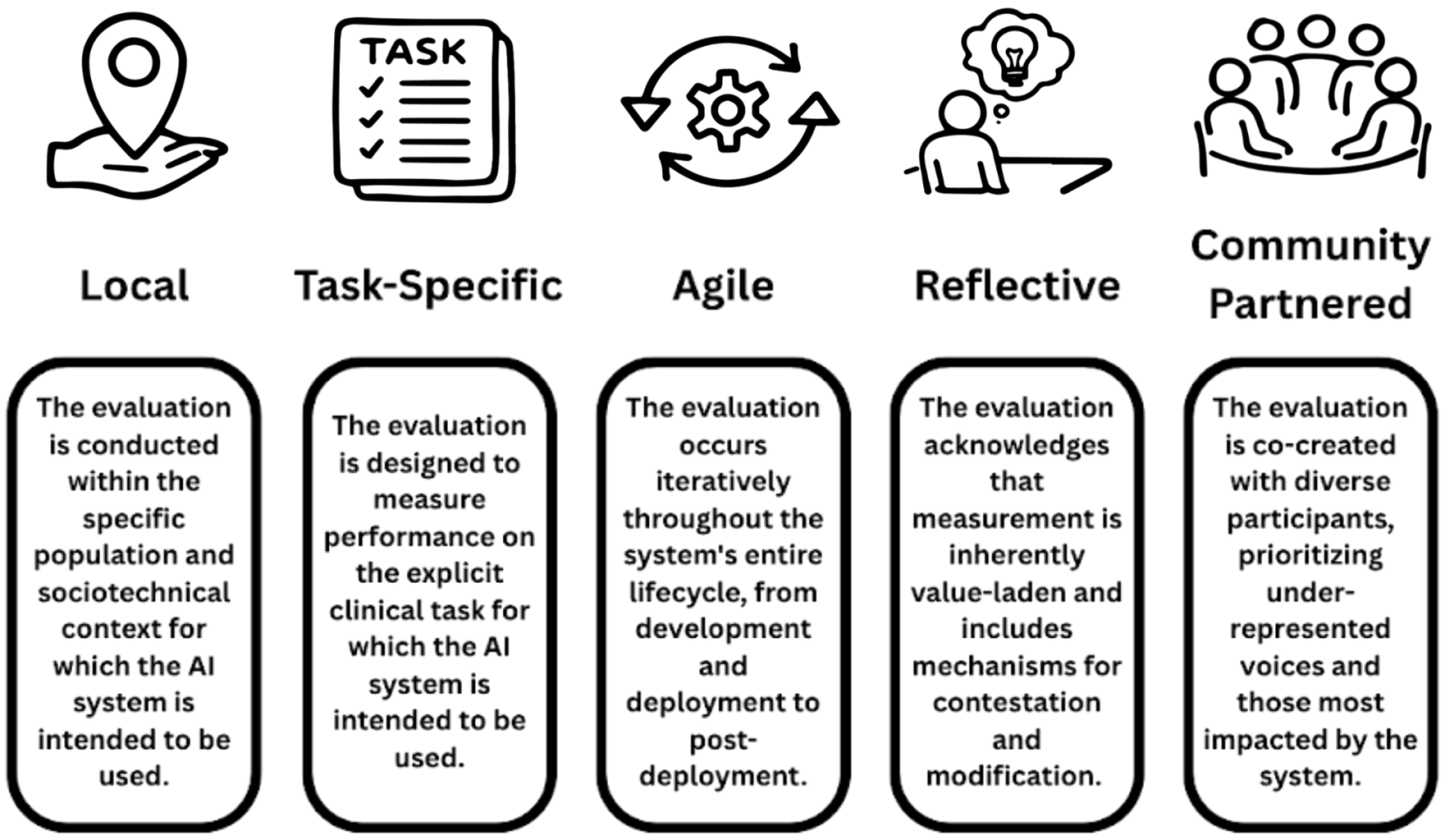
LTARC principles for context-aware evaluation of healthcare AI.

Meaningful evaluation of healthcare AI must be anchored in the specific clinical settings, populations, and use cases where systems are intended to be deployed (Fig 1) [14]. The premise that general-purpose models merit general-purpose evaluations fundamentally misunderstands how clinical needs, data distributions, and definitions of benefit vary across contexts [15]. Data shift between different populations referenced in model evaluation and implementation drives most AI failures in healthcare related to predictive performance [16]. Evaluation of a model using a different population than the one for whom it will be implemented can appear falsely successful. Epic’s sepsis model exemplified this when external validation revealed an AUROC of 0.63, far below advertised performance at the time of its initial evaluation [17].

**Fig 2:**
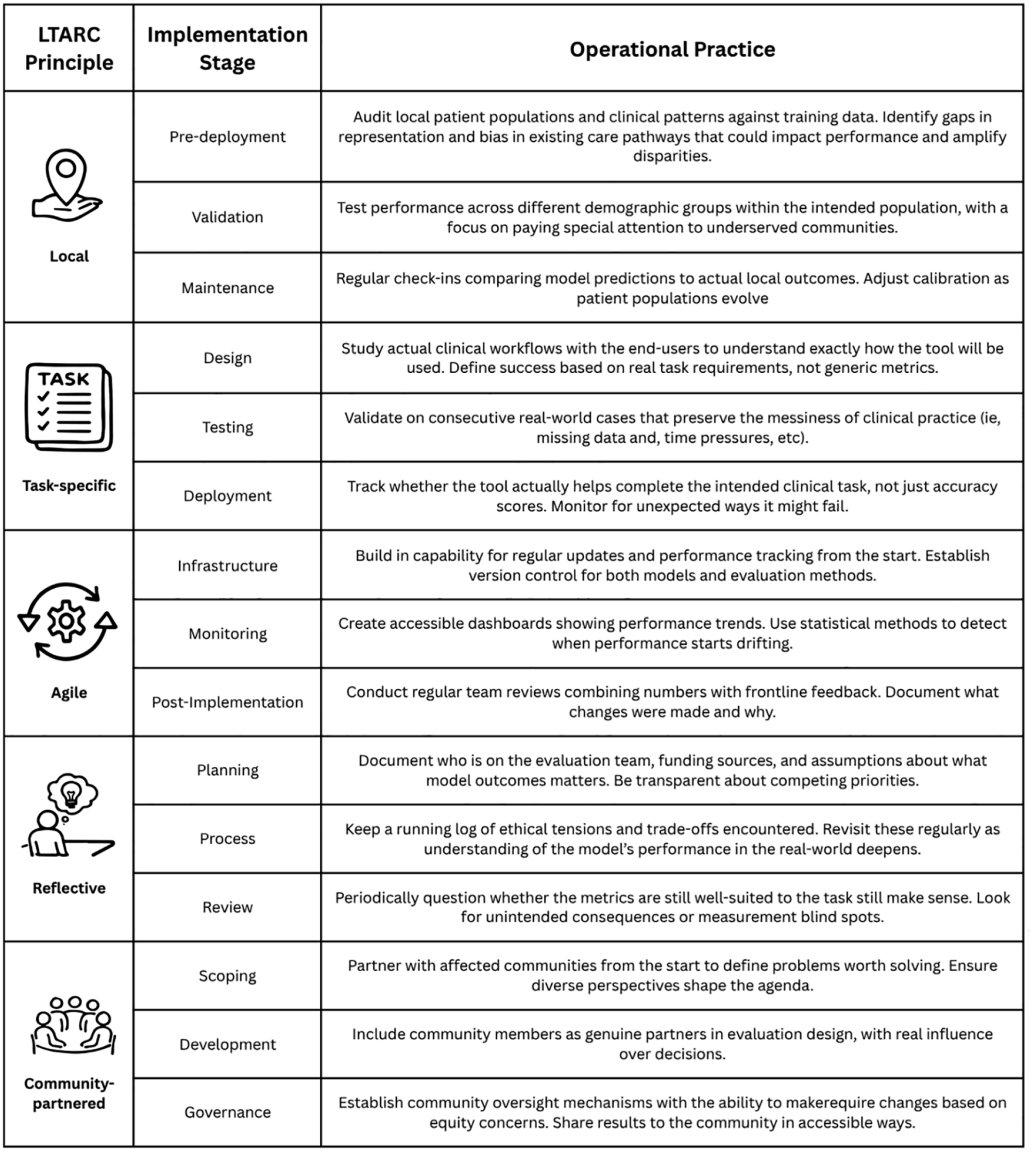
Operationalizing LTARC principles across the AI lifecycle.

Current evaluation practices reveal troubling disparities that are important to highlight. Among 2,425 US hospitals using predictive models, only 61% evaluate accuracy locally and just 44% assess bias [15]. In the same study, well-resourced health systems were found to have significantly higher evaluation rates than critical access, rural, and high Social Deprivation Index hospitals serving the most vulnerable populations. In practice, effective local evaluation may involve auditing local data distributions or recalibrating models using site-specific patient populations (Fig 2). The goal is not to abandon generalizability, but to establish local evaluation as the foundation for any broader claims. Even applications requiring wide deployment, such as public health surveillance systems must validate within specific contexts to ensure safety, efficacy, health system alignment, and equity.

Local evaluation imposes real costs, e.g., financial resources for validation studies, technical expertise for local data analysis, and adequate sample sizes for statistically meaningful assessment, that are inequitably distributed across health systems. Well-resourced academic centers can more readily conduct rigorous local validation than critical access hospitals or safety-net institutions serving the populations most vulnerable to algorithmic harm, creating a perverse situation where those least able to evaluate are most in need of protection. For rare conditions or presentations, local validation may indeed be statistically infeasible, requiring alternative approaches such as federated evaluation networks where similar institutions pool validation data, reliance on broader regional or national validation with explicit documentation of uncertainty when local prevalence differs, or deployment restrictions limiting AI use to well-validated common presentations while preserving human judgment for rare cases. However, we maintain that these practical constraints do not invalidate the Local principle but rather highlight structural inequities in healthcare AI governance: if local validation is too expensive or technically demanding for community hospitals, the solution is not to abandon contextual evaluation but to question whether AI systems should be deployed in settings lacking resources to evaluate them safely, to demand that vendors provide turnkey local validation tools rather than treating validation as purchaser responsibility, and to establish public infrastructure supporting local evaluation as a prerequisite for equitable AI deployment. The practical difficulties the reviewer identifies are real barriers that must be addressed through policy, funding, and shared evaluation infrastructure, and not reasons to retreat to decontextualized benchmarks that systematically disadvantage under-resourced settings.

### Task-specific: aligning evaluation with intended use

Evaluation frameworks must directly assess performance on the specific clinical tasks for which an AI model is intended to be used (Fig 1). Performance gaps are more likely when general-purpose models are applied to specific clinical tasks. One comprehensive evaluation using 2,400 real patient cases found that general-purpose LLMs were unable to reliably order diagnostic tests appropriately, follow instructions for a prolonged period of time, or produce consistent outputs across different prompt formulations [18]. While this evaluation examined earlier models like GPT-3.5 and Llama 2 70B, these task-specific failures likely persist even if the context window and number of training parameters substantially increase [19]. This task-specific imperative extends beyond diagnostic accuracy to encompass full clinical workflows and recommendations. Among 519 evaluated healthcare LLM studies, only 5% used real patient data for evaluation and merely 0.2% assessed performance on administrative tasks.

Most importantly, task-specific evaluation must ensure that predicted outcomes serve as valid proxies for meaningful clinical endpoints, rather than convenient measurable surrogates. For example, using healthcare utilization as a proxy variable for severity of illness systematically underestimates the needs of populations with low utilization as a result of disparate healthcare access, embedding inequity into the model’s very foundation [20]. This issue of construct validity becomes particularly important when the relationship between measurable labels and intended clinical concepts varies across patient populations. Task-specific evaluation must therefore move beyond generic displays of medical knowledge to assess whether models reliably perform their intended functions. Handling uncertainty, integrating clinical reasoning, and aligning with care protocols represent essential and distinct competencies requiring highly intentional evaluation (Fig 2).

### Agile: embracing continuous adaptation

Healthcare AI evaluation must evolve beyond static, one-time assessments to embrace continuous adaptation and facilitate iterative improvement (Fig 1). First, continuous evaluation mitigates model degradation, a persistent and well-documented challenge. One longitudinal study tracked four top-performing machine learning models across 1.83 million records over 2.5 years, finding gradual performance decline—most noticeable at one year—with significantly worse AUROC, accuracy, precision, and recall due to temporal shifts in patient characteristics [21]. Another study simulating model influence on care using 130,000 ICU admissions found that the intervention effect itself eroded predictive validity over time [22]. The root causes of AI performance degradation over time are multifaceted: temporal data drift, calibration drift, evolving protocols, population shifts, and inadequate detection/correction methods for retraining [21].

Continuous model evaluation presents responsiveness and recalibration challenges, for which a model’s agility is critical to responsible implementation [23]. Rapid evaluation responsiveness draws from agile software development practices, which have shown value in healthcare AI implementation by emphasizing incremental change, rapid feedback cycles, and adaptive planning [24]. Agile evaluations must extend beyond technical metrics to encompass clinical utility reassessments, monitoring not just declining predictive accuracy but whether models continue serving their intended purpose as contexts evolve (Fig 2).

### Reflective: acknowledging values and limitations

Evaluation is never value-neutral (Fig 1). Every metric chosen, dataset selected, and threshold established embeds assumptions about whose perspectives matter and what constitutes success. Prioritizing technical metrics such as efficiency, scalability, and accuracy often advances institutional priorities while potentially undermining patient-centered outcomes like dignity, trust, and reduced treatment burden [25]. These tensions between values widely vary between relevant groups. Community members may prioritize communication quality and preservation of human connection, while clinicians may value workflow integration and clinical utility [26]. Personal values resist technical optimization and demand intentional, transparent navigation of trade-offs throughout the evaluation.

Reflective evaluation requires structured interrogation of epistemological assumptions. Teams should critically consider and document evaluator backgrounds, funding sources, and prior beliefs about patient priorities or clinical workflows [27]. This critical self-examination must be embedded into the evaluation infrastructure itself, not appended as an afterthought. The WHO’s ethics framework for large multi-modal models exemplifies this approach, prompting developers to document guiding ethical frameworks and explicitly address value conflicts between community members [6]. Such reflection demands epistemic humility, thereby acknowledging the fundamental uncertainty inherent in such a vastly complex system where evaluations span technical performance, patient experiences, organizational dynamics, community impacts, and clinical workflows. Reflective evaluation cannot occur in isolation. It requires a diverse community transforming evaluation from a measurement exercise into a collective ethical practice (Fig 2).

### Community-partnered: centering affected voices

Finally, rigorous evaluation of healthcare AI must begin and end with the people it is meant to serve. This principle moves beyond the Local (focusing on data fit) and Reflective (examining evaluators’ values) principles by addressing fundamental questions of power, representation, and shared decision-making throughout the model lifecycle (Fig 1). Meaningful early and continuous involvement of all relevant actors (especially those from vulnerable and marginalized populations) is a nonnegotiable prerequisite for valid evaluation. We adopt the language of co-design and co-production to emphasize epistemic equality and shared authority in evaluation processes [28]. Community members are not ‘stakeholders’ consulted for input, nor ‘participants’ enrolled in research studies, but co-designers who hold genuine decision-making authority over evaluation criteria, success metrics, and deployment decisions. This framing recognizes communities as possessing irreplaceable knowledge about what constitutes benefit, harm, and acceptable risk in their specific contexts, and knowledge that technical experts cannot substitute for through proxy measures or demographic categories. Those most likely to be harmed by algorithmic bias must be centered in the design and execution of the evaluation process, as their lived experiences are essential for defining what constitutes a benefit or a harm across all stages of a model’s development and deployment.

Despite widespread agreement on the importance of community involvement in healthcare AI development, implementation remains rare. A scoping review in 2024 of 10,880 healthcare AI publications found that only 21 studies (0.2%) reported any amount of community involvement with just one study engaging community participants during the model design phase [29]. When perspectives regarding how to measure concepts like quality, bias, and harm differ across populations, evaluations that fail to account for that diversity necessarily misrepresent key concepts of interest across those populations [30]. Meaningful community engagement in the evaluation phase may involve co-identification of clinical pain points with community groups, negotiating data-sharing agreements that respect community ownership, including community participants on the design team itself, and establishing community advisor boards who retain veto power over model updates based on community-driven equity dashboards (Fig 2) [31]. Without authentic community voices, even evaluations incorporating all other LTARC principles remain fundamentally inequitable.

LTARC recognizes that community engagement can appropriately begin at different research phases depending on the study’s goals, resources, and stage of development. For exploratory research examining technical feasibility, such as developing novel algorithms using existing datasets, community engagement may focus on interpretation, governance, and deployment decision-making rather than initial problem scoping. However, as research transitions toward deployment, community validation becomes essential: Do the outcomes we’re optimizing actually matter to patients? Does ‘solving’ this technical problem improve care experiences or primarily serve institutional efficiency goals? The critical LTARC requirement is not that communities define all research questions, but that deployment decisions incorporate community perspectives on whether the ‘problem’ being solved aligns with patient priorities. This validation might occur at scoping for deployment-oriented initiatives, or during development/governance phases for exploratory work transitioning toward implementation. The key is ensuring that at some point before deployment, researchers ask communities: ‘We can build this, but should we?’

## Operationalizing the LTARC principles

Existing evaluation approaches, from reporting standards like CONSORT-AI [32] and TRIPOD-AI [33] to technical frameworks like the National Institute of Standards and Technology or NIST’s AI Risk Management Framework [34] and regulatory pathways like the U.S. Food and Drug Administration or FDA’s Good Machine Learning Practice [35], provide essential infrastructure for healthcare AI governance. These frameworks address critical questions of what to report, how to manage risk, and which regulatory gates to navigate. However, they largely assume that technical performance metrics validly represent clinical benefit and that context can be controlled for rather than centered. LTARC complements these existing frameworks by addressing epistemological gaps: questioning whether measured outcomes capture patient priorities, treating local context as foundational rather than secondary, requiring continuous adaptation rather than fidelity to validated interventions, and centering affected communities with decision-making authority rather than consultation roles. The goal is not to replace existing frameworks but to establish principles that guide their application toward genuinely patient-centered evaluation.

Rather than dismissing prior evaluation efforts, LTARC builds upon decades of rigorous work in performance metrics, fairness measurement, and clinical validation. The framework asks: once we have measured area under the receiver operating characteristic curve or AUROC or calibration or demographic parity, *what then?* Who interprets these metrics for which stakeholders? How do we monitor ongoing performance? Who is accountable when metrics degrade? What regulatory structures ensure appropriate use? And fundamentally, do our metrics actually measure outcomes patients care about? LTARC positions quantitative evaluation as an essential foundation, not an endpoint.

Operationalizing these principles requires fundamental shifts in how healthcare organizations, developers, and regulators approach AI evaluation (Fig 2). Organizations are encouraged to build infrastructure for local evaluation and continuous monitoring with early community involvement, treating evaluation as essential infrastructure prior to deployment rather than as a compliance checkbox. This approach demands evaluation partnerships from the outset by embedding monitoring capabilities, feedback mechanisms, and boundaries for local adaptation into system design. Finally, approaching evaluation with humility means acknowledging our knowledge limitations and confronting biases that perpetuate existing health disparities. Regulators should incentivize continuous, representative evaluation processes over one-time certifications while funders should prioritize patient-partnered innovations incorporating meaningful clinical outcomes.

We acknowledge that LTARC currently describes an aspiration rather than a reality. The gap between these principles and current practice reflects not individual failings but structural misalignment between responsible AI development and existing incentive systems. Well-intentioned researchers lack resources; profit-driven vendors lack motivation; health systems prioritize efficiency over equity. However, this gap is not immutable. Clinical trials, IRB review, and informed consent were once aspirational principles that became nonnegotiable requirements through regulatory action and professional norm-setting. The question is not whether healthcare AI can be developed responsibly under current structures, but whether we will collectively build the regulatory, economic, and institutional infrastructure to make responsible development the path of least resistance rather than heroic exception. LTARC provides a framework for what that infrastructure must accomplish. Converting aspiration to requirement demands advocacy from patients, clinicians, researchers, and communities most affected by algorithmic medicine. It requires regulatory bodies willing to impose standards that may slow deployment and reduce profits. It demands funders supporting unglamorous work like long-term monitoring over novel algorithm development. Most fundamentally, it requires rejecting the premise that healthcare AI development should be governed primarily by market forces and technical feasibility rather than by patient welfare and health equity.

### Use case: AI-assisted radiology interpretation in two deployment contexts

The same AI system deployed in two different contexts requires fundamentally different evaluation approaches reflecting local populations, clinical workflows, institutional priorities, and community needs (Table 1). A well-resourced academic medical center (left column) implements LTARC principles to ensure AI supports subspecialist decision-making, integrates with complex workflows, maintains academic standards, and serves diverse patient populations. A community hospital serving predominantly uninsured patients (right column) implements the same principles to ensure AI improves care access, supports general radiologists practicing without immediate subspecialist backup, reduces financial barriers to diagnosis, and responds to community-defined priorities. These contrasting operationalization scenarios demonstrate why standardized evaluation protocols cannot substitute for context-responsive, community-partnered assessment.

**Table 1:** LTARC principles applied to AI-assisted radiology interpretation.

LTARC principle	Well-resourced academic medical center	Community hospital serving predominantly uninsured patients
**LOCAL** (grounding in specific context)	• Validate using the institutional imaging archive with local protocol variations and scanner characteristics • Assess performance across specialty services (trauma, oncology, pediatrics) reflecting academic case mix • Calibrate confidence thresholds based on local prevalence of rare pathologies • Benchmark against subspecialty radiologist interpretations available on-site	• Validate using community patient demographics and disease prevalence patterns • Account for limited specialist availability and reliance on general radiologists • Assess performance on portable/lower-quality imaging common in resource-constrained settings • Evaluate impact on patients who delay care due to cost: does AI reduce unnecessary follow-up imaging expenses?
**TASK-SPECIFIC** (Aligning with intended clinical use)	• Evaluate AI as decision support for subspecialists (second reader, triage for urgent findings) • Assess integration with PACS^1^ workflow and structured reporting templates • Test performance on complex cases requiring correlation with prior studies and clinical history • Measure impact on radiologist efficiency without compromising diagnostic quality	• Evaluate AI as primary diagnostic support for general radiologists without immediate specialist backup • Assess whether AI recommendations increase confidence for radiologists practicing beyond their usual scope • Test performance on screening studies where AI may enable services previously unavailable • Measure impact on patient access—does AI enable same-day reads vs. delayed interpretations?
**AGILE** (continuous adaptation)	• Implement a real-time monitoring dashboard tracking performance by modality, body region, and indication • Establish monthly interdisciplinary review (radiologists, IT, quality) of flagged discrepancies • Maintain a rapid recalibration pipeline when a new scanner or protocol is introduced • Quarterly subgroup analysis by patient demographics and insurance status	• Monitor performance against community radiologist interpretations and available follow-up data • Establish an accessible feedback mechanism for radiologists to flag problematic AI recommendations • Adapt thresholds based on seasonal disease patterns and patient population shifts • Annual community health board review of AI impact on care access and patient outcomes
**REFLECTIVE** (acknowledging values and limitations)	• Document institutional priorities: efficiency, research opportunities, and maintaining academic reputation • Interrogate whether “productivity gains” benefit radiologists' well-being or increase corporate revenue • Examine the assumption that subspecialist availability makes AI “optional enhancement” vs. necessity • Assess whether AI performance metrics prioritize rare academic cases over common community diagnoses	• Document community priorities: affordable access, trust in care quality, reducing referral burden • Interrogate whether AI deployment serves patient access or substitutes for hiring additional radiologists • Examine the assumption that AI can safely replace subspecialist consultation for underserved patients • Assess whether efficiency gains translate to improved patient access or reduced hospital costs without benefit sharing
**COMMUNITY-PARTNERED** (Centering affected voices)	• Establish a patient advisory board including diverse socioeconomic backgrounds, not just educated/insured • Engage radiologists, referring physicians, and trainees in defining acceptable AI errors vs. unacceptable failures • Partner with patient advocates to define communication standards when AI contributes to diagnosis • Ensure institutional review board includes community representatives, not just academic physicians	• Co-design evaluation with uninsured patient advocates who understand barriers to follow-up care • Partner with community health workers to define whether AI reduces or increases care navigation burden • Engage safety-net clinic physicians in defining acceptable performance thresholds given limited alternatives • Establish a community health board with veto authority over deployment if AI compromises care quality or access

^1^PACS = Picture Archiving and Communication System.

### The value proposition: why bear the costs?

Implementing LTARC principles imposes real costs—financial resources for validation studies, time for community engagement, expertise for continuous monitoring, and institutional commitment to potentially uncomfortable reflexivity. These barriers are structural (misaligned incentive systems, resource inequities between well-funded and safety-net institutions), cultural (academic metrics privileging publication volume over community partnership, resistance to ongoing monitoring in resource-constrained clinical workflows), and financial (direct costs of local validation, community engagement infrastructure, and continuous monitoring systems). The question deserves to be addressed directly: why should healthcare systems, developers, or researchers bear these costs?

The case for LTARC rests on four complementary arguments. First, moral imperative: deploying AI systems in vulnerable populations without adequate context-specific evaluation and community partnership perpetuates rather than mitigates health disparities. When well-resourced academic centers can afford rigorous local validation while safety-net hospitals serving predominantly uninsured patients cannot, algorithmic systems systematically advantage the already-advantaged. LTARC principles are ethical prerequisites for equitable AI deployment, not optional enhancements for well-intentioned developers.

Second, risk mitigation: inadequate evaluation creates patient safety risks and institutional liability that dwarf LTARC implementation costs. Epic’s sepsis prediction model, which appeared successful in initial validation, demonstrated AUROC of 0.63 in external hospital settings—barely better than chance and potentially worse than clinical judgment alone. Institutions that deployed this system based on decontextualized validation exposed patients to substandard care and themselves to reputational and legal risk. The cost of one failed deployment—in patient harm, institutional credibility, regulatory scrutiny, and rebuilding trust—exceeds the investment in rigorous context-specific evaluation across multiple successful implementations.

Third, long-term efficiency: continuous monitoring and community partnership identify failures early, before they scale. The real expense is not ongoing evaluation but deploying ineffective or harmful systems at scale, then managing the fallout when harms materialize. Community engagement surfaces implementation barriers before launch, reducing costly post-deployment modifications. Continuous monitoring catches performance degradation while interventions remain manageable, avoiding catastrophic failures requiring complete system withdrawal. LTARC principles reduce the total cost of ownership by preventing expensive failures.

Fourth, regulatory inevitability: emerging frameworks from the FDA (Good Machine Learning Practice requiring ongoing monitoring), EU AI Act (mandating high-risk system oversight and stakeholder consultation), and WHO (emphasizing community engagement and context-specific validation) increasingly require LTARC-aligned practices. Institutions building evaluation infrastructure now will face lower compliance costs than those retrofitting systems to meet evolving regulatory standards. The question is not whether to implement LTARC principles but whether to do so proactively or reactively under regulatory pressure.

We acknowledge these arguments may not persuade actors prioritizing short-term profit maximization or publication metrics over patient benefit. This is precisely why we advocate for regulatory requirements making LTARC compliance nonoptional, reimbursement structures rewarding patient-centered outcomes over efficiency metrics, public funding mechanisms supporting community engagement and local validation infrastructure, and vendor accountability frameworks shifting evaluation burden from purchasers to developers. Individual researchers and well-intentioned health systems cannot bear these costs alone without systemic support.

The barriers to LTARC implementation are real and demand structural solutions: regulatory mandates, public infrastructure investment, academic incentive reform, and vendor accountability mechanisms. But the fundamental question is not whether healthcare systems can afford LTARC principles. It is whether patients can afford their absence. Every AI system deployed without adequate context-specific evaluation, without community partnership, without continuous monitoring, represents a decision that developer convenience and institutional efficiency matter more than patient safety and health equity. LTARC makes that decision explicit and demands justification.

## Conclusion

In evaluating healthcare AI, we argue that emphasis should not be on adding more frameworks to an already crowded landscape, but rather on embracing foundational principles that honor the complexity of clinical practice. The LTARC principles (Local, Task-specific, Agile, Reflective, and Community-partnered) offer a starting point for navigating the inherent tensions between technological capabilities, clinical impact, and community priorities. These principles are neither final nor comprehensive, but rightly offer a challenge to the paradigm of static benchmarks. Healthcare AI evaluations should begin to think beyond methodological optimization and rather consider which tools are deployed, how they evolve, who benefits, and who bears the risks. As healthcare AI becomes integral to clinical practice, our evaluation approaches must embody medicine’s core commitments: a commitment to continuous learning, epistemic humility, and above all, the imperative to do no harm.
